# Brain endothelial LRP1 maintains blood–brain barrier integrity

**DOI:** 10.1186/s12987-021-00260-5

**Published:** 2021-06-19

**Authors:** Steffen E. Storck, Magdalena Kurtyka, Claus U. Pietrzik

**Affiliations:** Molecular Neurodegeneration, Institute for Pathobiochemistry, University Medical Center of the Johannes Gutenberg University of Mainz, Duesbergweg 6, 55099 Mainz, Germany

**Keywords:** Blood–brain barrier integrity, Low-density lipoprotein receptor-related protein 1 (LRP1), P-glycoprotein/Abcb1 (P-gp), Tight junctions, Matrix metalloproteinases (MMPs), Cyclophillin A

## Abstract

The entry of blood-borne molecules into the brain is restricted by the blood–brain barrier (BBB). Various physical, transport and immune properties tightly regulate molecule movement between the blood and the brain to maintain brain homeostasis. A recent study utilizing a pan-endothelial, constitutive *Tie2-Cre* showed that paracellular passage of blood proteins into the brain is governed by endocytic and cell signaling protein low-density lipoprotein receptor–related protein 1 (LRP1). Taking advantage of conditional *Slco1c1-CreER*^*T2*^ specific to CNS endothelial cells and choroid plexus epithelial cells we now supplement previous results and show that brain endothelial *Lrp1* ablation results in protease-mediated tight junction degradation, P-glycoprotein (P-gp) reduction and a loss of BBB integrity.

## Background

Neuronal function requires tight regulation of the cerebral microenvironment, which is achieved through specialized brain barriers such as the blood–brain barrier (BBB) [1]. Dysfunction of these barriers lead to neuronal degeneration and cognitive decline [2]. A recent report demonstrated that global endothelial loss of the endocytic and cell signaling protein low-density lipoprotein receptor–related protein 1 (LRP1) utilizing a constitutive *Tie2-Cre* line results in increased brain penetration of blood-borne molecules such as IgG and fibrinogen, progressive neuronal damage and behavioral deficits in mice [3]. Nikolakopoulou and colleagues identified a cyclophilin A–matrix metalloproteinase (MMP)-9 pathway in the *Lrp1*-deficient endothelium underlying BBB impairment: Deletion of LRP1 elevates cyclophilin A levels, which increases metalloproteinase-9-mediated tight junction protein degradation which allows the paracellular brain penetration of blood proteins leading to neuronal damage. Notably, LRP1 gene therapy targeting the BBB partially reversed vascular leakage, neuronal damage and behavioral deficits in mice.

Whilst these findings have broad implications for understanding how loss of endothelial LRP1 contributes to brain pathology, some questions for the audience remain. It is incompletely described how transcellular passage of molecules contributes to brain leakage of blood-borne molecules. Tight junctions are not the only regulator of BBB permeability. The authors did not see any effects of *Lrp1* deletion on pericyte coverage or endothelial MFSD2a and GLUT1 levels, known modulators of transcellular transport processes [4, 5]. However, endothelial solute and adenosine triphosphate binding cassette (ABC) efflux transporters such P-glycoprotein (P-gp, also known as ABCB1 or MDR1) limit the entry of many xenobiotics and endogenous molecules that might damage neuronal cells [6–8]. ABC transporter expression is regulated by various modulators such as notch signaling, wnt/β-cantenin signaling, pregnane X receptor, or peroxisome proliferator-activated receptors (PPAR) signaling [8–10]. Notably, it has been shown that endothelial LRP1 is a coactivator of the nuclear receptor PPARγ and directly participates in gene transcription [11]. If the PPAR signaling co-activator LRP1 is missing, ABC transporter levels could be altered. So, it is possible that, in addition to the described paracellular leakage described by Nikolakopoulou and colleagues, transcellular passage is altered due to a change in efflux transporters such a P-gp. Therefore, the increased permeability in *Lrp1*^*lox/lox*^; *Tie2-Cre* described in the recent paper would only be the result of increased paracellular passage through a lack of tight junctional proteins.

The second question remains regards the specificity of the *Cre* mouse line that was used in the study: how does a constitutive and global deletion of *Lrp1* in all endothelial cells contributes to the brain-related findings described in Nikolakopoulou et al.? In the study, the authors used a pan-endothelial expression of the *Cre* (*Tie2-Cre*) that targets all peripheral and CNS vasculature during development as well as adulthood. However, LRP1 is expressed in all endothelial cells throughout the organism and is involved in many endocytic and cell signaling events during development as well as angiogenesis [12, 13]. Therefore, *Tie2-Cre*-mediated *Lrp1* deletion could have averse off targets effects that contribute to the described pathology. Interestingly, we collected data showing similar results regarding BBB leakage in mice using a conditional, tamoxifen-inducible *Slco1c1-CreER*^*T2*^ not targeting peripheral vasculature and allowing the time-specific deletion of LRP1 in the vasculature of the brain [14].

## Results

**Fig. 1:**
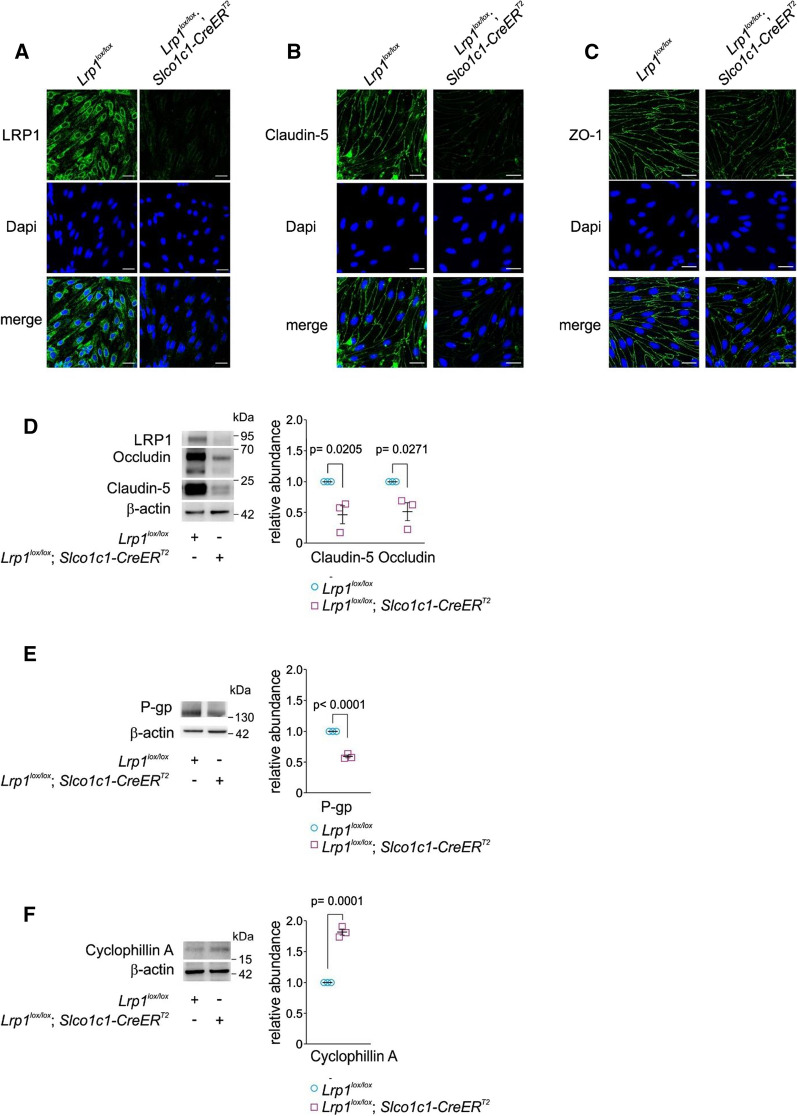
Reduced levels of tight junction proteins and P-gp after *Lrp1* CNS endothelial loss. Immunostaining for **A** LRP1, **B** claudin-5 and **C** ZO-1 in cultured endothelial cells of *Lrp1*^*lox/lox*^; *Slco1c1-CreER*^*T2*^ mice and *Lrp1*^*lox/lox*^ littermate controls. Scale bar: 20 µm. **D** Immunoblotting for occludin, and claudin 5. **E** cyclophillin A and **F** P-gp in isolated brain endothelial cells and their relative abundance compared with β-actin (loading control) of 2-month-old *Lrp1*^*lox/lox*^; *Slco1c1-CreER*^*T2*^ mice and *Lrp1*^*lox/lox*^ littermate controls. Mean ± SEM, n = 3 isolates/group. Significance was determined by Student’s t test

**Fig. 2:**
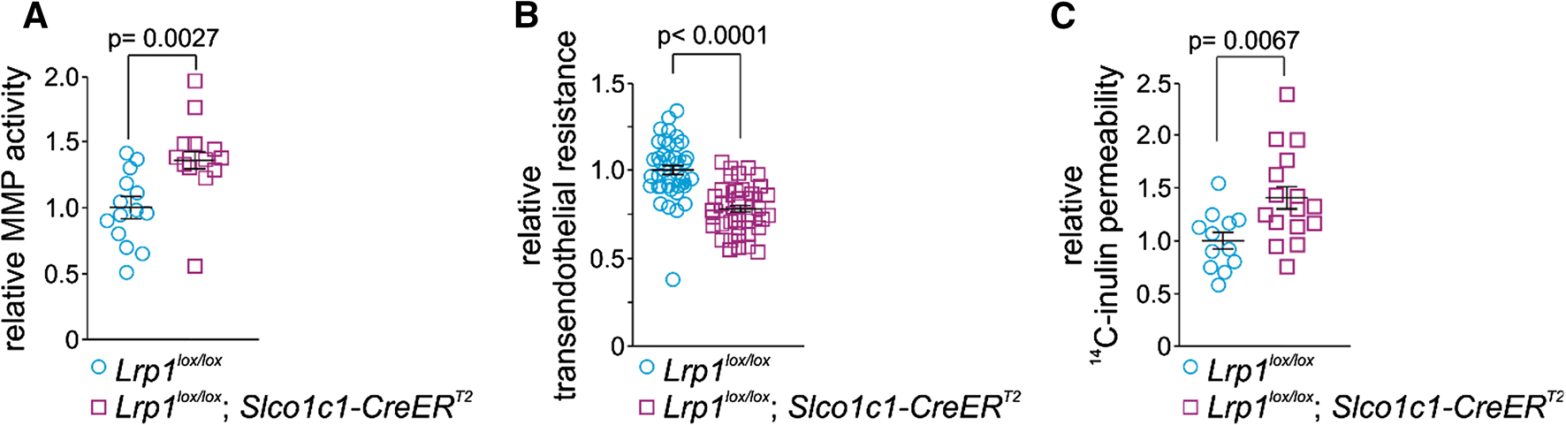
Enhanced MMP activity after *Lrp1* deletion increases permeability in cultured primary brain endothelial cells. **A** relative MMP activity, **B** transendothelial resistance and **C** ^14^C-inulin permeability of primary brain endothelial cells isolated from of 2-month-old *Lrp1*^*lox/lox*^; *Slco1c1-CreER*^*T2*^ mice and *Lrp1*^*lox/lox*^ littermate controls. Primary brain endothelial cells were cultivated on transwell inserts in the cellZcope device. Cells or supernatants were used for subsequent studies when confluent. TEER was 72.6 ± 2.53 for *Lrp1*^*lox/lox*^ and 56.69 ± 1.83 for *Lrp1*^*lox/lox*^; *Slco1c1-CreER*^*T2*^ endothelial cells. Mean ± SEM. **B** and **C** are biological replicates from 3 independent isolates. Significance was determined by Student’s t test

The solute carrier organic anion transporter *Slco1c1* is highly enriched in CNS vasculature over peripheral vasculature in mice [15]. Therefore, in *Slco1c1-CreER*^*T2*^ mice, where Cre-mediated gene excision is controlled by the *Slco1c1* promotor, genetic deletion of a gene of interest can be achieved by tamoxifen injection specifically in brain endothelium and choroid plexus epithelium [14]. Previously, we developed *Lrp1*^*lox/lox*^; *Slco1c1-CreER*^*T2*^ mice that allow the deletion of *Lrp1* in brain endothelial cells in adult mice. This strategy reduces potential off target side effects compared to pan-endothelial *Cre*-lines like *Tie2-Cre* by leaving peripheral vasculature unaffected. Moreover, in contrast to a constitutive, global *Tie2-Cre*-driven promoter [16], temporal induction of gene deletion in adult *Slco1c1-CreER*^*T2*^ mice can rule out any potential off target effects of *Lrp1* deletion during development. Using *Lrp1*^*lox/lox*^; *Slco1c1-CreER*^*T2*^ mice, we sought to investigate whether a knockout of *Lrp1* in CNS endothelial cells has any effect on barrier integrity of the BBB. When we cultured primary brain endothelial cells, we found that cells from *Lrp1*^*lox/lox*^; *Slco1c1-CreER*^*T2*^ mice showed lower levels of tight junctional proteins claudin-5 and zonula occludens-1 (ZO-1) compared to brain endothelial cells isolated from *Lrp1*^*lox/lox*^ littermates expressing LRP1 (Fig. 1A–C). Moreover, in freshly isolated brain endothelial cells of *Lrp1*^*lox/lox*^*; Slco1c1-CreER*^*T2*^ mice, claudin-5 and occludin protein levels were markedly reduced compared to littermate controls (Fig. 1D). At the same time, the levels of cyclophillin A, an activator of a MMP-mediated tight junction degradation pathway in endothelial cells [3], were significantly elevated (Fig. 1F). Utilizing primary brain endothelial cells from *Lrp1*^*lox/lox*^; *Slco1c1-CreER*^*T2*^ mice we found higher MMP activity (Fig. 2A) using a fluorogenic MMP substrate, lower transendothelial resistance (Fig. 2B) measured by impedance spectroscopy along with increased ^14^C-inulin permeability across an endothelial monolayer compared littermate control cells (Fig. 2C). These results suggested that elevated MMP activity in *Lrp1*^*lox/lox*^*; Slco1c1-CreER*^*T2*^ endothelial cells resulted in enhanced endothelial permeability due to tight junction degradation.

**Fig. 3:**
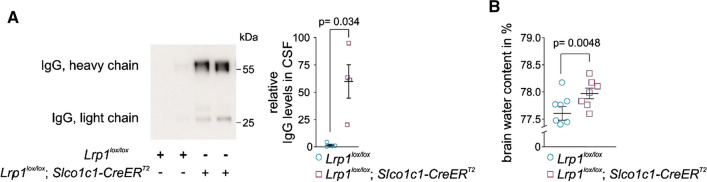
Enhanced BBB permeability after *Lrp1* CNS endothelial loss. **A** Immunoblotting and quantification for IgG in 3 µL cell- and blood-free CSF and **B** brain water content (calculated as [wet weight–dry weight] × 100/wet weight) in 20-month-old *Lrp1*^*lox/lox*^; *Slco1c1-CreER*^*T2*^ mice and *Lrp1*^*lox/lox*^ littermate controls. Mean ± SEM, n = 4 (in **A**) and 7 (in **B**) mice/group. Significance was determined by Student’s t test

**Fig. 4:**
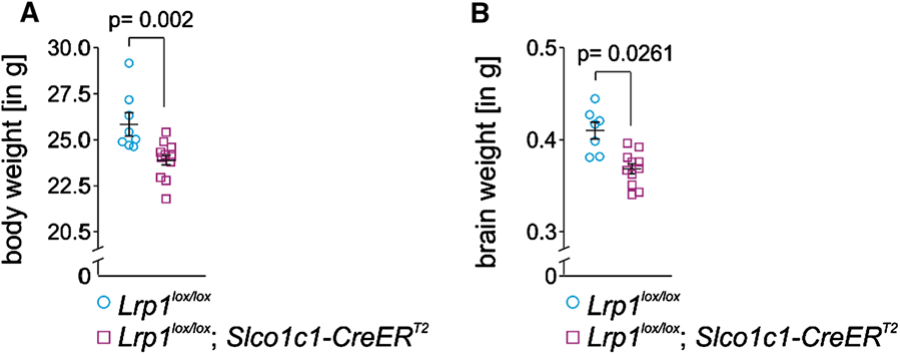
Reduced brain body and brain weight upon brain endothelial *Lrp1* deletion. **A** Brain (n = 7 + 10 mice, left to right) and **B** body weight (n = 8 + 11 mice, left to right) in 12-month-old *Lrp1*^*lox/lox*^; *Slco1c1-CreER*^*T2*^ mice and *Lrp1*^*lox/lox*^ littermate controls. Mean ± SEM. Significance was determined by Student’s t test

Disrupted tight junctions can lead to an extensive influx of hematogenous fluid and into the extravascular space leading to progressive elevation of brain water content and tissue swelling. Therefore, investigated whether the prolonged lack of LRP1 in brain endothelial cells has any effect on the brain penetration of endogenous IgG and water in aged mice. In 20-month-old mice, we detected substantially increased IgG levels in the cerebrospinal fluid (CSF) of *Lrp1*^*lox/lox*^*; Slco1c1-CreER*^*T2*^ mice compared to *Lrp1*^*lox/lox*^ littermates (Fig. 3A [17–19]. Moreover, *Lrp1*^*lox/lox*^*; Slco1c1-CreER*^*T2*^ showed a higher brain water content (Fig. 3B) than their *Lrp1*^*lox/lox*^ littermates suggesting that there is increased influx of water into the brain due to an opening of the BBB [17, 18]. Of note, along with altered tight junctions, we found P-gp decreased (Fig. 1E, also reported in [20]). It remains to be determined by future studies what the functional consequences on the loss of P-gp are for brain penetration of xenobiotics in *Lrp1*^*lox/lox*^*; Slco1c1-CreER*^*T2*^ mice. However, a recent study shows a direct effect on brain uptake of P-gp substrate rhodamine123 upon changes in P-gp transcript levels [21]. Besides P-gp, other ABC transporter levels could be affected due to the lack of PPARγ coactivator LRP1. This will remain a subject for subsequent studies. Collectively, these data suggest that BBB permeability is increased by paracellular and potentially transcellular mechanisms when endothelial LRP1 is absent. In *Lrp1*^*lox/lox*^*; Slco1c1-CreER*^*T2*^ mice both body and brain weight were significantly reduced when the animals were housed on a constant tamoxifen-supplemented chow (Fig. 4A, B) suggesting that lack of endothelial LRP1 impairs homeostasis and metabolism as also suggested by earlier studies [11].

Unexpectedly, as reported earlier, we did not find any differences in BBB integrity in *Lrp1*^*lox/lox*^*; Slco1c1-CreER*^*T2*^ at 8 months [22] measured by endogenous IgG in the brain or brain penetration of intravenously-injected sodium-fluorescein brain. In these experiments, the mice were housed on a normal chow lacking tamoxifen after an initial 7-days treatment with tamoxifen at 8 weeks of age. Given the massive damage occurring to CNS vasculature reported here and in the study by Nikolakopoulou and colleagues [3], we are now questioning, whether a long-term brain endothelial LRP1 deletion will prevail over time in a conditional system in older mice when the driving *Cre* is not constitutively expressed and only a single treatment of tamoxifen early in adulthood is applied as it was done in the study. It has been shown that vascular damage recruits bone marrow-derived endothelial progenitor cells (sometimes also referred as circulating angiogenic cells) from the periphery for vascular repair [23–25]. Different from a constitutive model as used by Nikolakopoulou and colleagues, a conditional model as used in our study could therefore regain gene expression over time by replacement of damaged endothelial cells with peripheral LRP1 expressing blood-circulating cells and therefore mask the initial effects of the initial knockout. To date, we have not tested whether long term tamoxifen deprivation after will result in a regain of LRP1 protein expression at the BBB. Further studies are needed to fully decipher the biological mechanisms underlying the effects seen in *Lrp1*^*lox/lox*^*; Slco1c1-CreER*^*T2*^mice.

Collectively, the data suggests that many of the results shown by Nikolakopoulou and colleagues using a constitutive, pan endothelial *Cre*, can be independently reproduced by using a conditional knockout model targeting CNS vasculature only. It seems that spatial and temporal control of endothelial LRP1 recapitulates the finding of a global, constitutive endothelial knockout. Both studies show, that LRP1 in brain endothelial cells is a critical regulator of BBB integrity and function. It remains to be demonstrated whether neuronal damage as described in Nikolakopoulou et al. are merely the results of increased paracellular influx of blood-borne molecules into the brain or whether altered transcellular movement of molecules, due to changes in ABC transporter expression, also contribute to brain pathology.

## Methods

### Mice

*Lrp1*^*lox/lox*^; *Slco1c1-CreER*^*T2*^ [22] and littermate *Lrp1*^*lox/lox*^ controls were housed under a 12-h light–dark cycle with water and rodent chow ad libitum. For all studies both sexes were used. Brain and body weight was analyzed with 12 months of age.

### Antibodies

Following antibodies were used for western blotting (WB) and immunocytochemistry (ICC): Rabbit anti-β-actin (A2066, Sigma-Aldrich, WB: 1:1000), Rabbit anti-claudin-5 (34-1600, Invitrogen, WB: 1:1000, ICC: 1:100), Mouse anti-occludin (33-1500, Invitrogen, WB: 1:1000), rabbit anti-zonula occludens 1 (ZO-1) (ICC: 1:100; ProteinTech, #21773-1-AP), H-241 rabbit anti-Mdr (sc-8313, WB: 1:1000 – detects MDR1&MDR3 mouse/rat/human), rabbit anti cyclophillin A (ab3563, Abcam,WB: 1:1000), 1704 rabbit anti-LRP1 (WB: 1: 10,000) and mouse monoclonal anti-LRP1 (11E2, ICC 5.2 μg/ml) were generated as described before [22, 26], HRP-conjugated donkey anti-mouse (715-035-151, Jackson Immuno Research, 1:5000), HRP-conjugated goat anti-rabbit (A5278, Sigma-Aldrich, WB: 1:10,000)., Alexa 488- or Alexa 546- conjugated secondary antibodies (1:1000; Abcam, #150077 and Thermo Fisher Scientific, A11018).

### Isolation and cultivation of primary mouse brain capillary endothelial cells

Primary mouse brain capillary endothelial cells were isolated from 8-week-old mice as described previously with minor modifications [22, 27]. In brief, mice were sacrificed by cervical dislocation, meninges were removed, cortices were pooled and mechanically dissociated, followed by a digest with a mixture of 0.75 mg/ml collagenase CLS2 (Worthington, Lakewood, NJ, USA) and 10 U/ml DNaseI (Sigma-Aldrich, Schnelldorf, Germany) in DMEM (Gibco, Darmstadt, Germany) at 37 °C on a shaker set at 1000 g for 1 h. The pellet was resuspended in 20% BSA-DMEM (w/v) and centrifuged at 1000 g for 20 min to remove myelin. The pellet was further digested with 1 mg/mL collagenase-dispase (Roche, Mannheim, Germany) and 10 U/mL DNAse in DMEM at 37 °C on a shaker for 1 h. Endothelial capillaries were separated on a 33% continuous Percoll (GE Healthcare, Munich, Germany) gradient, collected, and subjected to cell lysis or plated on 24-well transwell filters (pore size, 0.4 μm; surface area, 33.6 mm2; Greiner Bio-One) coated with 0.4 mg/mL collagen IV and 0.1 mg/mL fibronectin (both from Sigma-Aldrich, Schnelldorf, Germany). Cultures were maintained in DMEM supplemented with 20% plasma-derived bovine serum (First Link, Birmingham, UK), 100 U/mL penicillin and 100 μg/mL streptomycin, 2 mM L-glutamine (all from Gibco, Darmstadt, Germany), 4 μg/mL puromycin (Alexis, Loerrach, Germany) and 30 µg/ml endothelial cell growth supplement (Sigma-Aldrich, Schnelldorf, Germany) at 37 °C and 5% CO^2^.

For immunoblot analysis, isolated capillary fragments were solubilized in lysis buffer (50 mM TrisOH, 150 mM NaCl, 0.02% [w/v] NaN3, 1% [v/v] Nonidet P-40 supplemented with a cocktail of phosphatase and proteinase inhibitors [PhosStop, Complete, Roche Applied Science]). Homogenates were centrifuged for 20 min at 15,000 g, and the supernatant was collected. 10 µg of capillary lysate was separated on 4–12% Bis–Tris gels (NuPAGE™, Invitrogen) gels by SDS-PAGE, transferred onto nitrocellulose membranes (Millipore).

### Immunocytochemistry analysis

Confluent primary endothelial cells were washed 3 × with PBS, fixed with ice-cold methanol for 10 min and room-temperature acetone for 1 min. To block unspecific binding of antibodies, cells were incubated with 5% normal goat serum (Gibco) and 1% bovine serum albumin (Roth) for 60 min. Cells were incubated with primary antibodies: rabbit polyclonal zonula occludens 1 (ZO-1) (1:100; ProteinTech, #21773-1-AP), mouse monoclonal LRP1 (in-house made, 11E2, [22]) or rabbit polyclonal claudin-5 (1:100; Invitrogen, #34-1600) overnight at 4 °C. Next day cells were washed briefly, incubated with Alexa 488- or Alexa 546- conjugated secondary antibodies (1:1000; Abcam, #150077 and Thermo Fisher Scientific, A11018) and counterstained with DAPI. Images were acquired using LSM710 confocal microscope (Zeiss) and analysed with ImageJ software.

### Transendothelial electrical resistance and permeability studies

TEER and capacitance of cells were measured automatically every hour by impedance spectroscopy with the cellZscope device. When capacitance values were between 1.0 and 0.8 μF/cm2, indicating a confluent monolayer of cells, the TEER values were measured. Permeability to [C^14^]-inulin (Perkin-Elmer, Waltham, MA, USA) was analyzed as described previously [28].

### MMP activity

MMP activity was measured from equal volumes of cell-free supernatant from confluent endothelial cells grown on transwell filters as described above. 3 h after incubation with OMNIMMP® fluorogenic substrate (Enzo) at 37 °C, fluorescence of the cleaved substrate was measured at an emission/excitation wavelength of 280/360 nm according to the manufactures’ protocol.

### CSF isolation

Blood-free CSF of 20-month-old mice were taken by puncture of the cisterna magna as described previously [22]. After centrifugation at 900 × g for 10 min at 4 °C, 4 μl of cell-free CSF were diluted in water and mixed with equal amounts of 2 × RotiLoad (Carl Roth, Karlsruhe, Germany). The IgG protein levels in CSF were determined using a secondary anti-mouse antibody.

### Brain water content

Brain water content from 20-month-old mice was determined as described previously [18]. Mice were anesthetized, sacrificed by cervical dislocation, and the brain was immediately removed, weighed and then dried overnight at 100 °C. The dried brain was re-weighed and the brain water content calculated as (wet weight- dry weight) × 100/wet weight.

## Data Availability

All data generated or analysed during this study are included in this published article.

## Abbreviations

ABC: Adenosine triphosphate binding cassette
BBB: Blood brain-barrier
CSF: Cerebrospinal fluid
LRP1: Low-density protein receptor-related protein 1
MMP: Matrix metalloproteinase
PPAR: Peroxisome proliferator-activated receptors
P-gp: P-glycoprotein
ZO-1: Zonula occludens-1
